# Patient engagement in multimorbidity: a systematic review of patient-reported outcome measures

**DOI:** 10.3389/fpsyg.2024.1345117

**Published:** 2024-07-17

**Authors:** Serena Barello, Gloria Anderson, Caterina Bosio, Deirdre A. Lane, Donato G. Leo, Trudie C. A. Lobban, Caterina Trevisan, Guendalina Graffigna

**Affiliations:** ^1^EngageMinds HUB – Consumer, Food and Health Engagement Research Center, Università Cattolica del Sacro Cuore, Milan, Italy; ^2^Department of Psychology, Università Cattolica del Sacro Cuore, Milan, Italy; ^3^Fondazione Policlinico Universitario Agostino Gemelli IRCCS, Rome, Italy; ^4^Department of General Psychology, University of Padua, Padua, Italy; ^5^Liverpool Centre for Cardiovascular Science and Department of Cardiovascular and Metabolic Medicine, Faculty of Health and Life Sciences, University of Liverpool, Liverpool, United Kingdom; ^6^Department of Clinical Medicine, Aalborg University, Aalborg, Denmark; ^7^Arrhythmia Alliance, Stratford-upon-Avon, United Kingdom; ^8^Department of Medical Sciences, University of Ferrara, Ferrara, Italy; ^9^Faculty of Agriculture, Food and Environmental Sciences, Università Cattolica del Sacro Cuore, Cremona, Italy

**Keywords:** patient engagement, patient empowerment, assessment, measures, multimorbidity

## Abstract

**Background:**

People with multimorbidity are increasingly engaged, enabled, and empowered to take responsibility for managing their health status. The purpose of the study was to systematically review and appraise the psychometric properties of tools measuring patient engagement in adults with multimorbidity and their applicability for use within engagement programs.

**Methods:**

PubMed, Scopus, Web of Science, and PsycInfo were searched from inception to 1 July 2021. Gray literature was searched using EBSCO host-database “Open dissertation”. The reference lists of studies meeting the inclusion criteria were searched to identify additional eligible studies. The screening of the search results and the data extraction were performed independently by two reviewers. The methodological quality of the included studies was evaluated with the COSMIN checklist. Relevant data from all included articles were extracted and summarized in evidence synthesis tables.

**Results:**

Twenty articles on eight tools were included. We included tools that measure all four dimensions of patient engagement (i.e., engagement, empowerment, activation, and participation). Their psychometric properties were analyzed separately. Most tools were developed in the last 10 years in Europe or the USA. The comparison of the estimated psychometric properties of the retrieved tools highlighted a significant lack of reliable patient engagement measures for people with multimorbidity. Available measures capture a diversity of constructs and have very limited evidence of psychometric properties that are vital for patient-reported measures, such as invariance, reliability, and responsiveness.

**Conclusion:**

This review clarifies how patient engagement, as operationalized in measures purporting to capture this concept, overlaps with, and differs from other related constructs in adults with multimorbidity. The methodological quality of psychometric tools measuring patient engagement in adults with multimorbidity could be improved.

**Systematic review registration:**

https://www.crd.york.ac.uk/prospero/display_record.php?RecordID=259968, identifier CRD42021259968.

## 1 Background

In recent years the population aging has led to increase the proportion of people with multiple chronic conditions (i.e., multimorbidity) (World Health Organization, 2016). Risky habits and lifestyles, longer life expectancy, and improved health care have led one in three adults to suffer from multimorbidity (Divo et al., 2014). People with multimorbidity are individuals who live with two or more long-term conditions, one of which is either physical non-communicable disease or a mental health condition, or an infectious disease of long duration (World Health Organization, 2016). People with multiple long-term conditions are challenging to treat, are prone to experience complications such as readmissions, adverse drug interactions or death, and often require a great deal of social and psychological support (Divo et al., 2014; World Health Organization, 2016). Moreover, the risk of being diagnosed with multiple long-term conditions rises with age, is more common among women and in people of lower socio-economic status (Divo et al., 2014; World Health Organization, 2016). People with multimorbidity often report difficulties in managing their care pathways that are often designed to control and treat single health conditions (Dhere, 2016). Collectively this makes caring for these people, particularly challenging. Clinicians often struggle to find, personalize, and provide the best therapeutic pathways, interventions, and protocols for people with multiple long-term conditions (Smoth et al., 2013).

Simultaneously, Western culture has gradually shifted from a paternalistic care approach toward patient-centered care and participatory medicine (Weil, 2016; deBronkart, 2018). People with multimorbidity are increasingly engaged, enabled, and empowered to take responsibility for managing their health (Pushparajah, 2018). Health researchers and stakeholders have started to design, test, and implement engagement interventions for people with multiple long-term conditions, showing their positive effects on health outcomes, user satisfaction, communication between patients and health professionals, adherence to treatment regimes, and healthcare resources usage (Barello et al., 2016; Bombard et al., 2018). This has led to the increased relevance of the concept of patient engagement and its synonyms (e.g., patient empowerment, activation, participation) in the literature (Castro et al., 2016; Náfrádi et al., 2017). In the last ten years, several studies have attempted to clarify the concept of patient engagement (Barello et al., 2012; Fumagalli et al., 2015; Higgins et al., 2017). Menichetti et al. (2016) highlighted that many concepts in the current literature overlap with patient engagement, such as patient enablement, empowerment, activation, and participation, since all these concepts refer to people’ proactive role in the management of their own healthcare.

ln this context, the use of tools designed and tested to engage people with multiple long-term diseases should be promoted among clinicians. Despite longstanding calls for greater engagement of older adults with multiple long-term conditions in healthcare, current evidence suggests that this population can be successfully engaged (Dambha-Miller et al., 2021; Markle-Reid et al., 2021). People with multiple long-term diseases are a diverse group, ranging from relatively healthy, independent living individuals to very frail individuals with poor physical functioning and cognitive problems, which often can make patient engagement in healthcare a challenging goal.

Therefore, a systematic review of the available engagement measurement tools to evaluate and monitor the benefits of engagement programs for people with multiple long-term conditions may help clinicians improve their care pathways. In particular, the examination of reliability, validity, feasibility, and clinical utility of engagement tools is required to inform the selection of appropriate instruments and address how to effectively enhance engagement in individuals and groups. Thus, the main object of the study was to systematically review and appraise the psychometric properties of tools measuring patient engagement in adults with multimorbidity and their applicability for use within empowerment programs, with a distinct focus on tools which have been validated in people with cardiovascular diseases.

This systematic review has been guided by the following research questions:

• What tools have been developed and validated in the literature to measure patient engagement in adults with multiple long-term conditions?
• What are the best tools, in terms of methodological quality and goodness-of-fit, to measure patient engagement in adults with multiple long-term conditions?
• What are the main conceptual components of engagement tools to shape future engagement interventions in this population?

## 2 Methods

### 2.1 Design

This study was performed in two steps: (i) a systematic review of the psychometric properties of engagement scales and tools was performed; then (ii) the psychometric properties were assessed by following the COnsensus-based Standards for the selection of health Measurement Instruments (COSMIN) guideline for systematic reviews of patient-reported outcome measures (Mokkink et al., 2016; Prinsen et al., 2018). The study protocol was registered on PROSPERO (registration number: CRD42021259968).

### 2.2 Search methods

A search strategy was designed to retrieve published and unpublished studies measuring patient engagement in adults with long-term conditions (Supplementary Material 1). The search filters developed by the Oxford PROM group and Terwee et al. (2007) were then used to refine the search strategy. Pubmed, Scopus, Web of Science, and PsycInfo were searched from their inception to April 2024. Gray literature was checked on EBSCOhost-database “Open dissertation” to identify any other significant publications. A forward and backward snowball search was performed to identify additional relevant publications.

The following eligibility criteria were used to select studies: (a) concerned with the development and/or evaluation of measurement properties of instruments that measure engagement and all the related concept such as empowerment, patient participation and patient involvement; (b) including adults with long-term conditions, including either instruments validated on people with multiple long term conditions or validated on people with at least three different long-term conditions; (c) published or unpublished up to April 2024; and (d) available in a language accessible to the authors (English and Italian). Tools were excluded if they: (a) were based on a single item. The literature search was performed by one researcher and then two researchers independently screened the records based on the title and abstract against the inclusion criteria. For eligible studies, the full texts were retrieved, and the same two researchers independently evaluated the eligibility of each study, and decisions on study inclusion were based on joint agreement.

Data extraction was performed by two researchers and the following data was recorded: (i) author, year and country; (ii) language and setting; (iii) study design; (iv) key characteristics of study subjects; (v) name of measurement instruments and domains measured; (vi) number of items and (sub)scales and number and type of response categories; (vii) recall period and time needed for administration; (viii) scoring algorithm; (ix) mode of administration; (x) instructions given to those who complete the questionnaire; and (xi) licensing information and costs. The psychometric properties reported in the studies were independently extracted by four authors. Then, another researcher independently revised the data extracted for accuracy. Any changes were discussed, and a full agreement was reached among the researchers.

### 2.3 Quality appraisal

The COSMIN checklist (Mokkink et al., 2018) was used to evaluate the methodological quality of studies on measurement properties. The checklist uses a standardized descriptive framework to assess the measurement properties against quality markers in ten boxes (Mokkink et al., 2018). Each box includes a pool of items (from five to 18) scored on a four-point scale (from 1 ‘poor’ to 4 ‘excellent’). The overall score is obtained by taking the lowest score indicated by the items in the box: therefore, a final score is given for each psychometric property, ranging from ‘poor’ to ‘excellent’. The measurement property ‘criterion validity’ was not considered in this systematic review since no “gold standard” exists for measuring engagement; therefore, eight boxes were rated. One researcher underwent training in the use of the COSMIN guidelines while the second reviewer had previous experience in the field. The inter-rater agreement between the two reviewers for the quality appraisal was 86.36% (*k* = 0.79).

### 2.4 Synthesis

Included validation studies have been summarized according to the data extracted. The values of the psychometric properties evaluated, and the quality of the methodologies used in assessing these psychometric properties have been also summarized using a descriptive approach. The conceptual components for future engagement interventions were synthesized based on the conceptual framework underlying the single engagement tools.

## 3 Results

**FIGURE 1:**
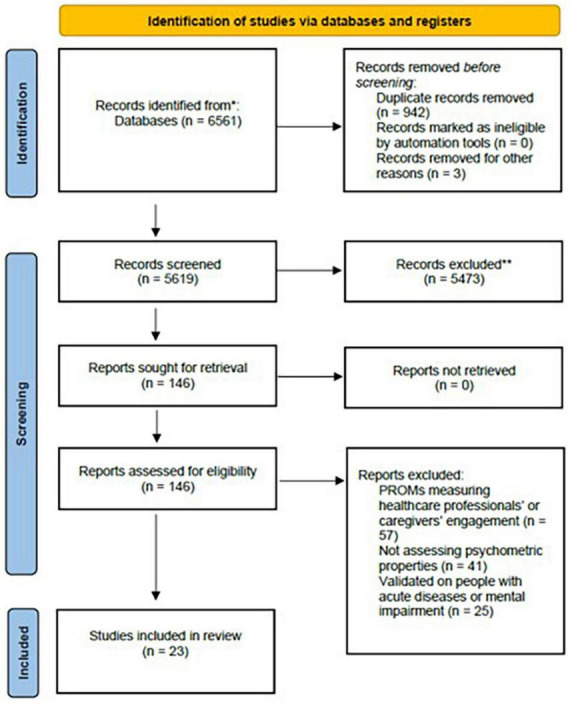
PRISMA flow diagram of the studies’ selection. *Consider, if feasible to do so, reporting the number of records identified from each database or register searched (rather than the total number across all databases/registers). **If automation tools were used, indicate how many records were excluded by a human and how many were excluded by automation tools.

The literature search produced 6,561 results, of which 942 duplicates were excluded. A total of 5,473 articles were excluded at the title and abstract screening stage, while other 123 articles were excluded at the full-text stage. Twenty-three articles (Hibbard et al., 2004; Glasgow et al., 2005; Wensing et al., 2008; Skolasky et al., 2011; Small et al., 2013; Koopman et al., 2014; Serrani Azcurra, 2014; Graffigna et al., 2015a,b; Schmaderer et al., 2015; Rademakers et al., 2016; Magallares et al., 2017; Moreno-Chico et al., 2017; Zhang et al., 2017; Cunha et al., 2019; Kosar and Besen, 2019; Usta et al., 2019; Zeng et al., 2019; Berg et al., 2020; Jerofke-Owen and Garnier-Villarreal, 2020) met the inclusion criteria describing eight families of tools as reported in Figure 1.

### 3.1 Study features

The main characteristics of the 23 articles (Hibbard et al., 2004; Glasgow et al., 2005; Wensing et al., 2008; Skolasky et al., 2011; Small et al., 2013; Koopman et al., 2014; Serrani Azcurra, 2014; Graffigna et al., 2015a,b; Schmaderer et al., 2015; Rademakers et al., 2016; Magallares et al., 2017; Moreno-Chico et al., 2017; Zhang et al., 2017; Cunha et al., 2019; Kosar and Besen, 2019; Usta et al., 2019; Zeng et al., 2019; Berg et al., 2020; Jerofke-Owen and Garnier-Villarreal, 2020) are reported in Table 1. The eight families of tools were categorized as those used to measure patient engagement in managing their own health and those used to measure patient engagement in managing their healthcare pathways (Table 1). Most studies validated or investigated the psychometric properties of the following tools: (i) the Patient Activation Measurement (PAM) (*n* = 10) (Hibbard et al., 2004; Skolasky et al., 2011; Graffigna et al., 2015b; Schmaderer et al., 2015; Rademakers et al., 2016; Moreno-Chico et al., 2017; Cunha et al., 2019; Kosar and Besen, 2019; Zeng et al., 2019); (ii) The Patient Assessment Care for Chronic Conditions (PACIC) (*n* = 3) (Glasgow et al., 2005; Wensing et al., 2008; Berg et al., 2020); and (iii) The Patient Health Engagement Scale (PHE-S^®^) (*n* = 5) (Graffigna et al., 2015a; Magallares et al., 2017; Zhang et al., 2017; Usta et al., 2019).

**TABLE 1:** Characteristics of the included studies.

Prom	References	Aim	Language	Final number of item and subscale	Type of response	Population, (%)	N	Age, mean (D) yrs	Setting
**Tools to measure patient engagement in managing their health**									
PHE-S^®^	Usta et al., 2019	To assess the psychometric properties of PHE-s in Turkish patients with chronic diseases.	Turkish	5 items	7-point Likert scale	Diabetes mellitus (33); hypertension (28.9); Cancer (21.9%); Cardiovascular disorders (18.4); chronic renal failure (13.2), rheumatologic disorders (9.7), Chronic obstructive pulmonary disease (7.9%)	114	55.9 (14.5)	Hospital
	Zhang et al., 2017	To translate the original, PHE-s into Chinese Mandarin and to evaluate its psychometric properties in a group of patients with chronic disease in China.	Chinese	5 items	7-point Likert scale	Hypertension (71), diabetes (29.2); cardiovascular disease (27.1.); cerebrovascular disease (13.3); Chronic obstructive pulmonary disease (10.4), cancer (2.4)	377	53.8 (11)	Primary care
	Magallares et al., 2017	To adapt the Patient Health Engagement scale to the Spanish population (S.PHE-s) following the guidelines for cross-cultural adaptations.	Spanish	5 items	7-point Likert scale	Hypothyroidism (16.9); Hypertension (12.3%); Crohn disease (7); asthma (6.8); migraine (6.5); diabetes (4.8), others	413	37.1 (11.8)	Primary care
	Graffigna et al., 2015a	To validate the patient Health Engagement Scale.	Italian	5 items	7-point Likert scale	Asthma (16.4); Hypertension (35.6), Cardiovascular disorder (15.3); chronic obstructive pulmonary disorder (4), cancer (21), fibromialgy (5.2), artritereumatoide (7.3); osteoarthritis (7.3); hypercholesterolemia (10.3); allergy (16.6)	430	51.3 (NR)	Hospital
	Changizi et al., 2023	To evaluate the psychometric features of the PHE-scale in Iranian patients with breast cancer	Iranian	5 items	7-point Likert scale	Long-term breast cancer	128	26–65 (8.11)	Hospital
PAM-13	Rademakers et al., 2016	To compare the psychometric properties in studies from the different countries and establish whether the scores on the PAM vary between the studies.	Danish; Dutch; German; Norwegian; English	13 items	Five possible responses, scoring ranging from 0 to 4	Adults with multiple chronic diseases from five different countries	5184	45–97*	Primary care & hospital
	Schmaderer et al., 2015	To investigate the psychometric properties of the PAM in patients with multimorbidity in the hospital setting.	English	13 items	Five possible responses, scoring ranging from 1 to 4	Adults discharged from an acute care facility with three or more chronic diseases	313	62.7 (15)	Hospital
	Skolasky et al., 2010	To determine the psychometric properties of PAM among multimorbid older adults and evaluate a theoretical, four-stage model of patient activation.	English	13 items	Five possible responses, scoring ranging from 1 to 4	Adults with an average of four multiple chronic diseases each	853	56.6 (12.9)	Primary care
	Kosar and Besen, 2019	To test the reliability and validity of a Patient Activation Measure.	Turkish	13 items	Five possible responses, scoring ranging from 0 to 4	Adults with multiple chronic diseases	130	56.7 (13.8)	Primary care
	Zeng et al., 2019	To assess the reliability and validity of the PAM13 in Chinese patients with hypertension and/or diabetes in a community management setting.	Chinese	13 items	Five possible responses, scoring ranging from 0 to 4	Hypertension (59.3), diabetes (17.9), hypertension and diabetes (22.8)	509	67.2 (8.9)	Primary care
	Moreno-Chico et al., 2017	To develop a European Spanish adaptation of the original PAM-13 and to examine its psychometric properties in a sample of chronic patients.	Spanish	13 items	Five possible responses, scoring ranging from 1 to 4	High blood-pressure (69.2); diabetes (66.3); dyslipidemia (49) and COPD (25.5)	208	65.8 (9.45)	Primary care
	Graffigna et al., 2015a	To validate a culturally adapted Italian Patient Activation Measure (PAM13-I) for patients with chronic conditions.	Italian	13 items & 1 dimensions	5-point Likert scale	Hypertension (20.2), Cardiovascular disorder (29.1), asthma (16.4) COPD (4) diabetes (16.2) cardiovascular disorder (29.1) oncology (21) fibromyalgia (5.2) osteoarthrosis (7.3) artritereumatoide (7.3); hypercholesterolemia (10.2) allergy (16.6)	529	53.0 (17.1)	Hospital
	Kerari et al., 2023	To determine the psychometric properties of the Arabic version of the Patient Activation Measure.	Arabic	13 items	Five possible responses, scoring ranging from 1 to 4	Adults with chronic conditions (40)	225	53 (12.5)	Primary care
	Zakeri et al., 2023	To translate the American versions of the PAM-13 into Persian and test the psychometric properties of the Persian version among chronic patients	Persian			Ischemic heart disease (IHD) (42,9), diabetes mellitus (DM) (12.6), hypertension (16.7), congestive heart failure (CHF) (10.3), chronic obstructive pulmonary disease (COPD) (9.4), other (8.2): chronic kidney disease (CKD), multiple sclerosis (MS), rheumatoid arthritis (RA), cancer, psychological disorders	438	62.21 (13.39)	Hospital
PAM-22	Paulo Silva Cunha and Dias, 2018	To adapt and validate the Patient Activation Measure (PAM22) in a sample of Brazilians with chronic diseases under outpatient monitoring.	Portuguese	22 items, 4 subscales	Five possible responses, scoring ranging from 1 to 4	Cancer (13.6) HIV/Aids (9.7) rheumatoid arthritis (9.9) systemic lupus erythematosus (6.8) Cron’s disease (7.8) diabetes (9.7) ulcerative RECTOCOLITIS (4.9) OBESITY (5.8) coronary insufficiency (8) chronic renal insufficiency (5.5) systemic arterial hypertension (9.6) cardiac failure (8.9) Cardiac failure (8.6%)	513	49.9 (14.6)	Primary care
	Hibbard et al., 2004	To develop a measure for assessing “activation,” and the psychometric properties of that measure.	English	22 items, 4 subscales	5-point Likert scale	Angina/heart problem (13), Hypertension (34) arthritis (38) chronic pain(25) depression (15) diabetes (11) lung disease (12) cancer (5) high cholesterol (30)	1515	45–54*	primary care
HES	Serrani Azcurra, 2014	To translate and adapt the Health Empowerment Scale (HES) for a Spanish-speaking older adults’ sample and perform its psychometric validation.	Spanish	8 items	5-point Likert Scale from 5 to 1	Hypertension (58.8) arthritis (40.3) diabetes (20.7) hyperlipidemia (17.1)	648	74.8 (11.6)	Primary care
Small’s scale	Small et al., 2013	To report on two empirical studies conducted to understand and measure empowerment in patients with long-term conditions in primary care.	English	8 items	4-point Likert scale	Diabetes (46.2) COPD (13.2) irritable bowel syndrome (21.8) arthritis (52.3) anxiety and depression (26.9) asthma (15.7) Coronary heart disease (16.8) Heart problems or high blood pressure (52.8)	197	62.8 (14.3)	Primary care
**Tools to measure patient engagement in managing their healthcare pathways**									
PACIC	Wensing et al., 2008	To develop and test a Dutch version of the PACIC questionnaire, a measure for patient reported structured chronic care.	Dutch	20 item & 5 subscales	Five-point response scale, ranging from 1 to 5	Adults with diabetes and/or COPD	165	68 (10.3)	Primary care
	Glasgow et al., 2005	To develop and validate the Patient Assessment of Chronic Illness Care (PACIC)	English	20 items & 5 subscales	Five-point response scale, ranging from 1 to 5	Adults with two different chronic conditions	266	64.2 (10.5)	Primary care
PPQ	Berg et al., 2020	To develop an instrument to measure patient participation in health care and to investigate the measurement properties of the Patient Participation Questionnaire (PPQ).	Danish	16 items & 4 subscales	4-point Likert Scale from 1 to 4	Hypertension (33) diabetes (13) cancer (5) depression (4)	378	<65	Hospital
PPET	Jerofke-Owen and Garnier-Villarreal, 2020	To develop and psychometrically test the Patient Preferences for Engagement Tool (PPET).	English	29 items	5-point Likert rating scale	Hypertension (34.7); heart disease (24.4); dyslipidemia (20.5); asthma (11); COPD (8.5) diabetes mellitus (22.7); arthritis (17.2); cancer (26.6)	308	58.2 (17.1)	Hospital
PRE-HIT	Koopman et al., 2014	To measure patient readiness to engage with health technologies among adult patients with chronic conditions.	English	28 items	4-point Likert scale	Hypertension (81), coronary artery disease (12) diabetes mellitus (39) heart failure (11)	200	54 (14)	Primary care

NR, not reported;

*age range in years.

The majority (78%) of the included studies were published in the last 10 years and included patients from 15 different countries, mainly North America (e.g., USA, Canada) and Europe (e.g., Denmark, Netherlands, UK, Italy) (Table 1). Six studies focused on the development and validation of these tools, while the others were adaptation, translation, and evaluation of their psychometric properties (Table 1). Among primary studies, the first data collection was performed in 2003 (Hibbard et al., 2004).

Overall, the number of participants involved ranged from 114 (Usta et al., 2019) to 5,184 patients (Skolasky et al., 2011). The response rate was only reported in ten studies and ranged from 48% (Hibbard et al., 2004) to 96.2% (Zhang et al., 2017). As shown in Table 1, tools were mainly validated among patients with diabetes (66%), hypertension and other cardiovascular morbidities (52%), or on people with multiple long-term conditions (23%). Most participants were female, and the mean age of participants varied from 37 (Magallares et al., 2017) to 74 years old (Small et al., 2013). The ethnicity of participants was only reported in eleven studies, and most participants were Caucasian. Most of the scales required patients to have a basic level of health literacy. Patients with cognitive or mental health problems were often excluded from the validation studies.

Almost all tools were validated either in hospitalized (35%) or in primary care populations (65%), except Skolasky et al. (2011) which employed data from both settings. All the included tools were self-report questionnaires. Few studies reported the completion time and ranged from less 7 min (Glasgow et al., 2005) to 12 min (Usta et al., 2019).

The number of evaluated psychometric properties ranged from two to six (Table 2). The most commonly assessed properties were structural validity and internal consistency. Only two studies evaluated measurement error (Hibbard et al., 2004; Graffigna et al., 2015a). None of the included studies evaluated measurement variance. However, given that the items included are a manifestation of different underlying constructs, these properties were evaluated individually for each group of tools (Table 2).

**TABLE 2:** Quality assessment of the included studies.

Instru-ment	References	Internal consistency	Reliability	Content validity	Structural validity	Hypotheses testing		Cross-cultural validity	Floor and/or ceiling effect
		α *Cronbach*	*ICC*	*S-ICV*	*Variance explained%, methods*	*Hypotheses*	*sub-groups*	*DIF analyses and forward-backward*	
PHE-s	Graffigna et al., 2015a	0.87	0.95	NA	χ2 = 10.98, CFI = 0.981, RMR = 0.018, RMSEA = 0.059	Invariance in the two subsamples divided by gender	By age and educational level	DIF backward-forward	Small floor effect (range 1.7–4.5%) moderate ceiling effect (range 27.6–55%)
	Magallares et al., 2017	0.85.	NA	NA	χ2 = 1.88, df = 4, *p* = 0.75; CFI = 0.99, RMR = 0.01, GFI = 0.99, RMSEA = 0.05	Correlations with life satisfaction, medicine adherence behavior, anxiety, depression	By gender	Multigroup analyses forward-backward	No severe floor or ceiling effect
	Zhang et al., 2017	0.89	0.52–0.79.	0.92	χ2 = 6.65, df = 4, *p* = 0.156; (CFI = 0.983, SRMR = 0.014, GFI = 0.979, RMSEA = 0.067	Positive correlation with patient activation and medication adherence	NA	NA forward-backward	No severe floor or ceiling effect
	Usta et al., 2019	0.80	0.61	0.89	CATPCA and Rasch analysis (varied 0.62 to 1.14)	NA	NA	NA forward-backward	NA
	Changizi et al., 2023	NA	NA	0.81	CATPCA and Rasch analysis (varied 0.658–0.932)	NA	NA	NA forward-backward	NA
PPET	Jerofke-Owen and Garnier-Villarreal, 2020	>0.7	NA	0.8	EFA = 45%, χ2 (309) = 453.35, CFI = 0.892, TLI = 0.878, RMSEA = 0.056, 90% CI [0.045, 0.067], SRMR = 0.125, gamma-hat = 0.933, gamma-hatadj = 0.918.	NA	By age, comorbidities, educational level, health perception	MULTI group comparisons forward-backward	NA
PRE-HIT	Koopman et al., 2014	>70	0.60–0.85	Face validity	NA	NA	NA	NA backward-forward	NA
PPQ	Berg et al., 2020	0.89.	NA	NA	RMSEA = 0.043, CFI = 0.98; TLI = 0.98	NA	NA	NA backward-forward	Strong ceiling effect (range 34–94%)
SDM-Q-9	Scholl et al., 2012	0.92	0.68	Face validity	NA	Correlation between OPTION and SDM-Q-9	NA	NA backward-forward	Low variance due to ceiling effects and floor effects
HES	Serrani Azcurra, 2014	α = 0.89	0.92	0.98	CFI, GFI and NNFI ≥ 0.90, and RMSEA ≤ 0.06; χ2(634) = 5425.72; *p* < 0.001; KMO = 0.890	Correlations between the HES total and item scores and the General Self Efficacy Scale, Swedish Rheumatic Disease Empowerment Scale and Making Decisions Empowerment Scale	NA	NA backward-forward	Floor and ceiling effects were small (<20%)
Small’s scale	Small et al., 2013	0.82	NA	NA	EFA = 45.7%	Hypothesize relationships with overall empowerment (or individual dimensions) based on existing theory or empirical data (self-efficacy; gender; patient enablement; quality of chronic care; age; ethnicity; level of education; etc.)	By comorbidities, gender, age, ethnicity, living arrangements, education, current work, depression, general health, and self-efficacy	Multi group comparisons backward-forward	NA
PACIC	Tušek-Bunc et al., 2014	0.93	Spearman correlation	NA	NA	NA	NA	NA forward-backward	NA
	Wensing et al., 2008	0.71–0.83	>0.70	NA	CFA = 70% KMO = 0.844; Bartlett’s test of spherity *p* = 0.000	Higher PACIC scores positively correlated to both patients’ perceived enablement after the latest visit to the GP and to patients’ overall evaluations of general practice.	NA	NA forward-backward	Several items might have floor or ceiling effects.
	Fan et al., 2017	0.96	NA	NA	CFA = 74% RMSEA estimate of 0.09; CFI, 0.91; NFI, 0.90; and NNFI, 0.89.	NA	NA	NA forward-backward	Floor and ceiling effects (range from 1.8 to 2%)
	Iglesias et al., 2014	NA	NA	NA	RMSEA < 0.08, WRMR < 0.1.00, CFI > 0.97	Correlation with demographic variable	By age, gender, education, comorbidities, annual blood pressure, weight and lipid measure	Multi group comparisons forward-backward	Floor effect (range from 7 to 67%) & ceiling effect (range from 4 to 46%)
	Glasgow et al., 2015	0.84	Test-retest reliability	Expert panel	NA	The PACIC and its scales would (a) generally not be related to patient demographics (e.g., gender, age, education) but (b) would be related to disease characteristics (e.g., number of comorbid conditions). The PACIC would be moderately related to, but not redundant, with measures of primary care and patient activation.	NA	NA backward-forward	No items had ceiling effect
PAM-13	Rademakers et al., 2016	0.80–0.88	Test-retest reliability	NA	NA	NA	NA	NA forward-backward	NA
	Schmaderer et al., 2015	0.88	NA	0.91	χ2 = 5 400.41, df 5 65, p.0.01.; SRMR = 0.087, RMSEA = 0.08 CFI = 0.89	PAM scores would have (a) an inverse relationship with depression, (b) a positive relationship with physical functional status and health care quality, and (c) no relationship with number of comorbidities or severity of illness.	By depression, functional status, and comorbidities	Multi group comparisons forward-backward	NA
	Skolasky et al., 2011	0.87	NA	NA	KMO = 0.96	Higher PAM scores are related to greater adherence to desirable health-related behaviors, higher functional status, and better health care quality. Patients’ level of activation is not correlated with their number of comorbid conditions. Negative correlation between the PAM and comorbid conditions.	NA	NA forward-backward	NA
	Stepleman et al., 2010	NA	NA	NA	CFA	Correlation with MSSE, BDI-II and MS QOL, lower depression, and higher well-being	By age, educational level	Multi group comparisons forward-backward	NA
	Zeng et al., 2019	0.92	NA	NA	χ2 = 139.3, df = 59, *P* < 0.001, RMSEA = 0.060, CFI = 0.957	NA	NA	NA forward-backward	Floor effect (range 1.8–5.2%) and ceiling effect (range 21.4–28.1)
	Eyles et al., 2020	0.92	NA	NA	χ 2 = 3901.0644, 3927 - 5 degrees of freedom, *P* = 0.61 (Kaiser-Meyer-Olkin value = 0.88 and Bartlett’s Test of Sphericity χ2 = 1404.0, df 78, *p* < 0.001	Moderate correlations between DASS and AQoL scores with PAM-13. Weak correlations (between PAM-13 and HOOS/KOOS ‘Pain’ and ‘Function in daily living’ subscale scores.	NA	DIF analysis forward-backward	No floor or ceiling effect
	Maindal et al., 2009	0.89	NA	NA	CFA = 43.2%	NA	NA	DIF analysis forward-backward	Floor effect was small (range 0.6–3.6%), but the ceiling effect was above 15% for all items (range 18.6–62.7%).
	Graffigna et al., 2015a	0.88	NA	NA	χ2 = 2129.7, df = 78, *p* < 0.001; Kaiser-Mayer-Olkin measure of sampling adequacy was equal to 0.89.	NA	NA	DIF analysis forward-backward	Small floor effect (range 1.7–4.5%) and a moderate ceiling effect (range 27.6–55.0%).
	Kapoor and Singh, 2020	0.84	NA	NA	NA	NA	NA	NA forward-backward	NA
	Kosar and Besen, 2019	0.81	0.98	NA	x2/df: 1.59, RMSEA: 0.071, CFI: 0.96, NNFI: 0.95, Kaiser Meyer Olkin coefficient was.75 and Barlett test was x2: 646.870; p: 0. 000.	NA	NA	NA forward-backward	NA
	Moreno-Chico et al., 2017	NA	NA	NA	Data showed a fit to the Rasch model	Correlation between self-efficacy, quality of life, visits to the emergency room and number of hospitalizations	NA	DIF analysis forward-backward	NA
	Ngooi et al., 2016	0.86	NA	NA	CFA = 77%	Correlation with depression and self-efficacy	NA	DIF analysis forward-backward	All items had a small floor effect, but nine out of 13 items had a ceiling effect larger than 15%.
	Laranjo et al., 2018	NA	NA	NA	The Rasch dimension explained 39.1% of the variance in the data.	NA	NA	DIF analysis forward-backward	no floor or ceiling effects.
	Hashim et al., 2020	0.87	NA	Face validity	EFA = 60% KMO value was 0.86 and the *p*-value was <0.0001 for Bartlett’s test of sphericity.	NA	NA	NA forward-backward	small floor effect (range 0–3.1%) and a moderate ceiling effect (range 5.4–26.9%)
	Kerari et al., 2023	McDonald’s omega 0.80	0.31 (item 2) to 0.57 (item 11)	NA	χ2 = 76.76, df = 51, *p* < 0.01; TLI = 0.94; CFI = 0.96; RMSEA = 0.04 [90% CI = 0.02–0.07	NA	NA	Multi group comparisons forward-backward	N/A
	Zakeri et al., 2023	0.88	0.96	0.91	EFA χ2 = 1265.85, df = 78, *p* < 0.001 KMO = 0.84 CFA χ2/d.f. = 1.82, RMSEA = 0.077, SRMR = 0.055, GFI = 0.91, CFI = 0.97, IFI = 0.97, NNFI = 0.96, PNFI = 0.70)	NA	NA	Multi group comparisons forward-backward	The floor effect was 5.2% (ranging from 2.3 to 10.3%), but the ceiling effect was 26.19% (ranging from 17.3 to 33.7%).
PAM-22	Cunha et al., 2019	NA	0.26–0.64	NA	Rasch model	No relationship between activation, gender, and age of the participants. Positive correlation between activation and time of diagnosis of the chronic disease	NA	NA forward-backward	NA
	Hibbard et al., 2004	0.87	Test retest reliability	Assessed by expert panel	Rasch model	Those with higher activation would be more likely to engage in specific self-care and preventive behaviors. Further, those with higher activation who have a specific chronic disease should be more likely to engage in the self-care behaviors specific to their condition (e.g., exercising to control arthritis pain). Similarly, it was hypothesized that those with higher measured activation should engage in other health “consumeristic” behaviors, such as seeking relevant health care information, being persistent in getting clear answers from providers, and using comparative performance information to make health care choices. Those with more activation would indicate less fatalism about their future health.	NA	NA backward-forward	NA

#### 3.1.1 Tools to measure patient engagement in managing their health

Five tools to measure patient engagement in managing their health were retrieved (Table 1).

The Patient Health Engagement Scale (PHE-S^®^) is a patient self-administered short psychometric questionnaire developed to measure the level of patient engagement in their healthcare function (Graffigna et al., 2015a). It consists of five items measured on a 7-point Likert scale, that allows patients to easily mirror their current emotional states and illnesses experience. The PHE-S^®^ has a robust theoretical foundation since it was developed from the Patient Health Engagement model (Graffigna et al., 2015a). Currently, six versions of this scale are available: Italian (Graffigna et al., 2015a); English (Graffigna et al., 2015a); Turkish (Usta et al., 2019); Spanish (Magallares et al., 2017); Chinese (Zhang et al., 2017); Persian [XXX]. Across these tools, the psychometric properties remain the same as the original version (Table 2), demonstrating the consistency of PHE-S^®^. All the validation studies tested the internal consistency of the tool. Structural validity was evaluated using the Categorical Principal Component Analysis (CATPCA), a confirmatory factor analysis (CFA) and a RASCH model (Table 2). Reliability was evaluated in three studies (from acceptable to very good), while cross-cultural validity was assessed in two (Table 2). All the PHE-S psychometric properties were judged as good or adequate. The only exception was the reliability of the Turkish version which was judged as doubtful (Table 2).

The Patient Activation Measure (PAM) (Hibbard et al., 2004) is a well-known tool to assess patients’ knowledge, skills, and confidence for managing their health. There are currently two versions of the PAM, the original 22-item (PAM-22) and the 13-item short form (PAM-13). The PAM measures patient activation on a 0–100 scale, and the patients’ responses are measured on a 5-point Likert scale. Several translations and validations of the PAM are available (Table 1), as well as the original version developed by Hibbard et al. (2004). The PAM shows different judgments of its psychometric properties among its validations: in some of the studies, the PAM demonstrated good construct validity, reliability, and internal consistency overall, in others the judgment is doubtful or inadequate (Table 2). However, the PAM is the only patient activation measures retrieved that has been validated in a wide range of chronic or multimorbid populations (Table 1).

The Health Empowerment Scale (HES) is a survey that measures patients’ self-management skills and decision-making abilities (Serrani Azcurra, 2014). The HES was adapted from the Diabetes Empowerment Short Form Scale (DES-SSF) and has 8 items measured on a 5-point Likert scale. The HES shows good internal consistency, construct validity and adequate reliability (Table 2). Small floor and ceiling effects were reported (Table 2). Its content validity and theoretical conceptualization were judged as doubtful since the HES has no real underlying conceptual model. Other studies are needed to evaluate the consistency of the HES psychometric properties.

Small et al. (2013) developed a short questionnaire to measure empowerment in patients with long-term conditions (primarily diabetes, irritable bowel syndrome, coronary heart disease, or chronic obstructive pulmonary disease). It has 8 items measured on a 4-point Likert scale. Its structural validity appears to be doubtful, and no content validity was provided (Table 2).

#### 3.1.2 Tools to measure patient engagement in managing their healthcare pathways

Four tools measuring patient engagement in healthcare were identified.

The Patient Assessment of Care for Chronic Conditions (PACIC) is a survey that measures specific actions that chronic patients report they have experienced in the healthcare system (Glasgow et al., 2005). The PACIC was developed from the Patient Centered model and has five subscales, measuring patients’ activation, delivery system experience, goal setting, problem-solving, and coordination involvement. Five studies utilizing the PACIC were retrieved (Table 1). The PACIC is a 20-item questionnaire, and it uses a 5-point response scale, with higher scores indicating better quality of care. Similar to the PAM, the various PACIC validation studies report different judgments of its psychometric properties (Table 2). The PACIC content validity has been assessed by Glasgow et al. (2005) and was rated as inadequate. Its’ structural validity was judged as very good only by two studies (Table 2). PACIC reliability was only assessed by three studies with two deeming its reliability as inadequate or doubtful.

The Patient Participation Questionnaire (PPQ) is an instrument developed to measure patient participation in their treatment and care (Berg et al., 2020). It has been validated in patients with multi-morbidity, where one-third of the sample were patients with hypertension (Berg et al., 2020). The PPQ is a short questionnaire with 16 items and four subscales, measured on a 4-point Likert scale. The PPQ has a good internal consistency, but its structural validity has been judged as doubtful, and no measures of its reliability have been provided yet (Table 2).

The Patient Readiness to Engage in Health Internet Technology (PRE-HIT) is a tool developed to measure the likelihood of using health information technology among patients with chronic conditions (Koopman et al., 2014). The PRE-HIT focuses on the measurement of patients’ engagement in specific conditions and 28 items measured on a 4-point Likert scale. Only its content validity, internal consistency and reliability were reported (Table 2).

The Patient Preferences for Engagement (PPET) tool was developed to assess patients’ preferences for engaging in healthcare (Jerofke-Owen and Garnier-Villarreal, 2020). The PPET was designed to inform the planning and delivery of individualized healthcare. The PPET consists of 29 items weighted with a 5-point Likert scale. No PPET composite score has been computed yet. The content validity was judged doubtful, while its reliability, structural validity, and internal consistency were rated as adequate or very good (Table 2). Other studies are needed to further evaluate the consistency of the PPET psychometric properties.

#### 3.1.3 Conceptual components for future engagement interventions

According to the synthesis of the conceptual models or frameworks behind the tools included in this review, we extracted eight main conceptual components to be considered for future patient engagement interventions. The conceptual components are emotional adjustment, self-efficacy, self-management, health literacy, shared decision making, collaborative goal setting, proactive communication with the care teams, and problem solving (Table 3).

**TABLE 3:** Components of engagement interventions for patients diagnosed with multiple chronic diseases.

Domain	Tool	Pillars for patient engagement interventions
**Patient engagement**		
	PHE-s	Emotional adjustment, proactive communication with the care team
	PPET	Health literacy, self-efficacy
**Patient activation**		
	PAM-13	Shared decision-making, health literacy, self-efficacy, self-management, goal setting, problem solving
	PAM-22	Shared decision-making, health literacy, self-efficacy, self-management, goal setting, problem solving
**Patient participation**		
	PACIC	Collaborative goal setting, problem solving, self-efficacy
	PRE-HIT	health literacy, self-efficacy, emotional adjustment
	PPQ	Shared decision making, self-efficacy
	SDM-Q-9	Shared decision making
**Patient empowerment**		
	HES	Shared decision making, self-efficacy, self-management skills, health literacy
	Small’s scale	Emotional adjustment, shared decision making, self-management

Emotional adjustment, mainly related to the “patient engagement” domain, - refers to the patients’ ability to cope with the diagnosis and to elaborate their own role in the disease management. Self-management and self-efficacy – mainly related to the “patient activation domain” - are two well-known components of engagement interventions and refer to patients’ ability to effectively recognize their needs and act proactively to fulfill them. Health literacy, mainly linked to the “patient empowerment” domain, refers to patients’ knowledge and ability to understand information provided by the healthcare providers or caregivers about the disease and treatment journey. Also shared decision making and proactive communication are common conceptual components of engagement measurement tools. Indeed, shared decision making – which is mainly related to the “patient participation” domain - is essential in making them able to proactively manage their disease by enabling an open dialogue with the healthcare team about therapeutic choices and strategies. Collaborative goal setting and problem-solving, mainly related to the patient are crucial skills that make patients able to effectively plan self-care activities and to engage in proactive behaviors toward their disease management.

## 4 Discussion

This systematic review retrieved eight different tools that measure patient engagement in people with multiple long-term diseases. The tools were analyzed separately, based on the construct they measured. Half of the tools retrieved focused on measuring patient engagement as the process of emotional adjustment and the acquisition of motivation to manage their disease or as a general process of acquisition of a higher level of power. The other half measured people’s ability to take an active part in their consultations with healthcare professionals. Overall, the structure of the instruments was heterogeneous, as were their psychometric properties. Many tools only partially described their psychometric properties, with few outlining their theoretical foundation. The best psychometric properties were reported by the PAM^®^ (Hibbard et al., 2004) and the PHE-S^®^ (Graffigna et al., 2015a), which are the most tested and cross-culturally validated measures of patient engagement in managing their health to date.

Most of the tools retrieved were developed and/or adapted in the last 10 years, highlighting the growing importance of the concept of patient engagement in healthcare. The tools were tested mainly in populations with diabetes or hypertension. This is not surprising given the mean age of people with long-term conditions (Busse et al., 2010) and the importance of engaging with these people to help them achieve a suitable quality of life (Yen and Lin, 2018; Søgaard et al., 2021). Most instruments were short (<15 items) and had a short completion time (less than 10 min). The psychometric properties most often measured and reported were internal validity, content validity and construct validity. Many tools which showed a good theoretical foundation and reliability (Table 2), lacked a formal assessment of their structural validity. It is important that future studies further clarify the construct validity of these tools. Floor and ceiling effects were reported with some tools, and this may be problematic as the response scale of these instruments was all measured using Likert scales. Only three tools (PAM, PACIC, and PHE-S^®^) were tested in more than two different populations. This highlights the importance of increasing the dissemination of the concept of engagement and its measurement tools across healthcare conditions and especially in developing countries.

None of the identified tools measured both patient engagement in managing their own health and the healthcare pathways. This may be due to the lack of consensus on a unique definition of patient engagement (Barello et al., 2012; Fumagalli et al., 2015; Higgins et al., 2017). Patient engagement is a construct that in the literature overlaps with other psychological constructs such as activation, participation, and empowerment. However, even if many of these concepts are strongly intersecting (e.g., patient engagement and patient empowerment), others clearly measure different aspects of the process of engagement (e.g., patient participation). This problem was originally highlighted by Fumagalli et al. (2015) and almost 7 years later remains unresolved. The development of a single tool that measures all the different constructs underlying the concept of patient engagement may be an effective way to ease the process of measuring engagement.

To our knowledge, only one previous review has focused on measuring the concept of patient engagement in healthcare. Jerofke-Owen et al. (2020) limited their review on tools measuring patients’ preferences for engagement in healthcare; however, they did not systematically retrieve and evaluated also the tools measuring patients’ engagement in managing their own health. While this approach may increase accuracy in the analysis of the finding, given the lack of clarity on the concept of engagement it could also limit the ability to synthesize the concept’s use in the literature and lead to the loss of many valuable tools. Instead, we choose to use an inclusive approach to gain a deeper understanding of all the tools available to measure the concept of patient engagement.

This review allowed us to reflect on the components that should characterize engagement interventions in the future. The conceptual models and frameworks of the engagement tools are characterized by components such as emotional adjustment, self-efficacy, self-management, health literacy, shared decision making, collaborative goal setting, proactive communication with the care teams, and problem-solving. Some of these components (e.g., shared decision making, and proactive communication with the care team) are particularly important to identify the best care pathways for people with multiple chronic conditions. Others instead (e.g., emotional adjustment, self-efficacy, self-management) are necessary to guarantee that people with multiple chronic conditions are confident and able to partake in complex decisions on prognosis, treatment options and prioritizing care driven by their own perspective on what is acceptable, feasible or meaningful. These findings suggest that future engagement interventions should consider all these components to be effective. Current literature on patient engagement intervention for people with multiple long-term conditions is very heterogeneous (Søgaard et al., 2021). This diversity in the evidence base challenges the ability to draw robust conclusions and the increasing interest in patient engagement in the last 10 years in Europe and America sets the stage for reflection.

This review has some limitations. Firstly, while there are many different related concepts of engagement, some central terms might be lacking. Therefore, we excluded some concepts, for instance, self-care, patient adherence, or patient compliance although they have been used as related concepts of engagement. From our perspective, these concepts are outcomes of engagement. We chose the concepts which have in recent years been used as describing the active role of patients in healthcare (Fumagalli et al., 2015; Magallares et al., 2017), assuming they had an up-to-date view of related concepts. Secondly, some measures were rather new, and their validation process may be still ongoing. Lastly, it is possible that some relevant articles written in languages other than English or Italian may have been missed.

## 5 Conclusion

This systematic review highlights the need for a more comprehensive measure of patient engagement which includes all its related concepts (i.e., patient empowerment, patient activation, patient participation) and addresses all the possible components of patient engagement (i.e., emotional adjustment, self-efficacy, self-management, health literacy, shared decision making, collaborative goal setting, proactive communication with the care teams, problem-solving). Despite policy interest and initiatives relating to patient engagement, there is limited evidence to support the reliability and validity of existing tools and for the specific application to people with multiple long-term conditions. Moreover, retrieved studies often lack cross-cultural validation of the measures. This is particularly relevant as research suggests that there are ethnic differences in illness perception and management (Hillier, 1991; Lip et al., 2002). Future research could usefully develop a definitive more comprehensive measure of patient engagement.

## Data availability statement

The raw data supporting the conclusions of this article will be made available by the authors, without undue reservation.

## Author contributions

SB: Writing – review and editing, Writing – original draft, Methodology, Investigation, Data curation. GA: Writing – review and editing, Writing – original draft, Methodology, Investigation, Data curation. CB: Writing – review and editing, Writing – original draft, Project administration, Investigation. DAL: Writing – review and editing. DGL: Writing – review and editing. TL: Writing – review and editing, Supervision, Conceptualization. CT: Writing – review and editing. GG: Writing – review and editing, Writing – original draft, Supervision, Methodology, Conceptualization.

## References

[B1] Barello S. Graffigna G. Vegni E. (2012). Patient engagement as an emerging challenge for healthcare services: Mapping the literature. *Nurs. Res. Pract.* 2012:905934. 10.1155/2012/905934 23213497 PMC3504449

[B2] Barello S. Triberti S. Graffigna G. Libreri C. Serino S. Hibbard J. (2016). eHealth for patient engagement: A systematic review. *Front. Psychol.* 6:2013. 10.3389/fpsyg.2015.02013 26779108 PMC4705444

[B3] Berg S. K. Færch J. Cromhout P. F. Tewes M. Pedersen P. U. Rasmussen T. B. (2020). Questionnaire measuring patient participation in health care: Scale development and psychometric evaluation. *Eur. J. Cardiovasc. Nurs.* 19 600–608. 10.1177/1474515120913809 32324044

[B4] Bombard Y. Baker G. R. Orlando E. Fancott C. Bhatia P. Casalino S. (2018). Engaging patients to improve quality of care: A systematic review. *Implement Sci.* 13:98. 10.1186/s13012-018-0784-z 30045735 PMC6060529

[B5] Busse R. Blumel M. Scheller-Kreinsen D. Zentner A. (2010). *Tackling chronic diseases in Europe. Strategies, interventions and challenges*. Copenhagen: WHO Regional Office for Europe.

[B6] Castro E. M. Van Regenmortel T. Vanhaecht K. Sermeus W. Van Hecke A. (2016). Patient empowerment, patient participation and patient-centeredness in hospital care: A concept analysis based on a literature review. *Patient Educ. Couns.* 99 1923–1939. 10.1016/j.pec.2016.07.026 27450481

[B7] Changizi M. Cheraghiyan B. Mohamadian H. Ghorbani Kalkhajeh S. Salmanzadeh S. Maghsoudi F. (2023). The association between covid-19 information channels and preventive behaviors in Southwest of Iran: Application of Protection Motivation Theory (PMT). *Health Educ. Health Promot.* 11 1001–1008.

[B8] Cunha C. M. da Cunha D. Manzato R. O. Nepomuceno E. da Silva D. Dantas R. (2019). Validation of the Brazilian version of the patient activation measure 13. *J. Nurs. Meas.* 27 97–113. 10.1891/1061-3749.27.1.97 31068494

[B9] Dambha-Miller H. Simpson G. Hobson L. Roderick P. Little P. Everitt H. (2021). Integrated primary care and social services for older adults with multimorbidity in England: A scoping review. *BMC Geriatr.* 21:674. 10.1186/s12877-021-02618-8 34861831 PMC8642958

[B10] deBronkart D. (2018). The patient’s voice in the emerging era of participatory medicine. *Int. J. Psychiatry Med.* 53 350–360. 10.1177/0091217418791461 30114957

[B11] Dhere A. (2016). Managing complex long-term conditions and multimorbidity. *Clin. Med. (Lond).* 16 545–547. 10.7861/clinmedicine.16-6-545 27927819 PMC6297343

[B12] Divo M. J. Martinez C. H. Mannino D. M. (2014). Ageing and the epidemiology of multimorbidity. *Eur. Respir. J.* 44 1055–1068. 10.1183/09031936.00059814 25142482 PMC4918092

[B13] Eyles A. Gibbons S. Montebruno P. (2020). *Covid-19 school shutdowns: What will they do to our children’s education?* London: London School of Economics and Political Science.

[B14] Fan L. H. Gao L. Liu X. Zhao S. H. Mu H. T. Li Z. (2017). Patients’ perceptions of service quality in China: An investigation using the SERVQUAL model. *PloS one* 12:e0190123. 29272312 10.1371/journal.pone.0190123 PMC5741236

[B15] Fumagalli L. P. Radaelli G. Lettieri E. Bertele’ P. Masella C. (2015). Patient Empowerment and its neighbours: Clarifying the boundaries and their mutual relationships. *Health Policy* 119 384–394. 10.1016/j.healthpol.2014.10.017 25467286

[B16] Glasgow R. E. Neta G. Carpenter C. R. Grimshaw J. M. Rabin B. A. Fernandez M. E. (2015). A framework for enhancing the value of research for dissemination and implementation. *Am. J. Public Health* 105 49–57. 25393182 10.2105/AJPH.2014.302206 PMC4265905

[B17] Glasgow R. E. Wagner E. H. Schaefer J. Mahoney L. D. Reid R. J. Greene S. M. (2005). Development and validation of the Patient Assessment of Chronic Illness Care (PACIC). *Med. Care* 43 436–444. 10.1097/01.mlr.0000160375.47920.8c 15838407

[B18] Graffigna G. Barello S. Bonanomi A. Lozza E. (2015a). Measuring patient engagement: Development and psychometric properties of the Patient Health Engagement (PHE) Scale. *Front. Psychol.* 6:274. 10.3389/fpsyg.2015.00274 25870566 PMC4376060

[B19] Graffigna G. Barello S. Bonanomi A. Lozza E. Hibbard J. (2015b). Measuring patient activation in Italy: Translation, adaptation and validation of the Italian version of the patient activation measure 13 (PAM13-I). *BMC Med. Inform. Decis. Mak.* 15:109. 10.1186/s12911-015-0232-9 26699852 PMC4690217

[B20] Hashim H. A. Maulood M. F. Rasheed A. M. Fatak D. F. Kabah K. K. Abdulamir A. S. (2020). Controlled randomized clinical trial on using Ivermectin with Doxycycline for treating COVID-19 patients in Baghdad, Iraq. *MedRxiv.* [Preprint] 10.1101/2020.10.26.20219345

[B21] Hibbard J. H. Stockard J. Mahoney E. R. Tusler M. (2004). Development of the Patient Activation Measure (PAM): Conceptualizing and measuring activation in patients and consumers. *Health Serv. Res.* 39(4 Pt 1) 1005–1026. 10.1111/j.1475-6773.2004.00269.x 15230939 PMC1361049

[B22] Higgins T. Larson E. Schnall R. (2017). Unraveling the meaning of patient engagement: A concept analysis. *Patient Educ. Couns.* 100 30–36. 10.1016/j.pec.2016.09.002 27665500

[B23] Hillier S. (1991). The health and health care of ethnic minority groups. *A: SCRAM-BLER, G. Sociology as applied to medicine.* Londres: Baillière Tindall, 146–159.

[B24] Iglesias E. B. del Río E. F. Calafat A. Hermida J. R. F. (2014). Attachment and substance use in adolescence: A review of conceptual and methodological aspects. *Adicciones* 26 77–86. 24652402

[B25] Jerofke-Owen T. A. Garnier-Villarreal M. (2020). Development and psychometric analysis of the patient preferences for engagement tool. *Nurs. Res.* 69 289–298. 10.1097/NNR.0000000000000423 31977839

[B26] Jerofke-Owen T. Garnier-Villarreal M. Fial A. Tobiano G. (2020). Systematic review of psychometric properties of instruments measuring patient preferences for engagement in health care. *J. Adv. Nurs.* 76 1988–2004. 10.1111/jan.14402 32350898

[B27] Kapoor A. Singh E. (2020). Empowering smart cities though community participation a literature review. *Smart Cities—Opportun. Chall.* 2019 117–125.

[B28] Kerari A. Almalki M. Bahari G. Alharbi M. F. (2023). Validation of the Arabic version of the Patient Activation Measure (PAM-13) for Application within the primary healthcare context in Saudi Arabia. *Healthcare* 11:3090). 38063658 10.3390/healthcare11233090 PMC10706281

[B29] Koopman R. J. Petroski G. F. Canfield S. M. Stuppy J. A. Mehr D. R. (2014). Development of the PRE-HIT instrument: Patient readiness to engage in health information technology. *BMC Fam. Pract.* 15:18. 10.1186/1471-2296-15-18 24472182 PMC3916695

[B30] Kosar C. Besen D. B. (2019). Adaptation of a patient activation measure (PAM) into Turkish: Reliability and validity test. *Afr. Health Sci.* 19 1811–1820. 10.4314/ahs.v19i1.58 31149012 PMC6531939

[B31] Laranjo L. Dunn A. G. Tong H. L. Kocaballi A. B. Chen J. Bashir R. (2018). Conversational agents in healthcare: A systematic review. *J. Am. Med. Inform. Assoc.* 25 1248–1258. 30010941 10.1093/jamia/ocy072 PMC6118869

[B32] Lip G. Y. Kamath S. Jafri M. Mohammed A. Bareford D. (2002). Ethnic differences in patient perceptions of atrial fibrillation and anticoagulation therapy: The West Birmingham Atrial Fibrillation Project. *Stroke* 33 238–242. 11779916 10.1161/hs0102.101817

[B33] Magallares A. Graffigna G. Barello S. Bonanomi A. Lozza E. (2017). Spanish adaptation of the patient health engagement scale (S.PHE-s)in patients with chronic diseases. *Psicothema* 29 408–413. 10.7334/psicothema2017.75 28693715

[B34] Maindal H. T. Sokolowski I. Vedsted P. (2009). Translation, adaptation and validation of the American short form Patient Activation Measure (PAM13) in a Danish version. *BMC Public Health* 9:209. 10.1186/1471-2458-9-209 19563630 PMC2712471

[B35] Markle-Reid M. Ganann R. Ploeg J. Heald-Taylor G. Kennedy L. McAiney C. (2021). Engagement of older adults with multimorbidity as patient research partners: Lessons from a patient-oriented research program. *J. Comorb.* 11:2633556521999508. 10.1177/2633556521999508 33796472 PMC7975523

[B36] Menichetti J. Libreri C. Lozza E. Graffigna G. (2016). Giving patients a starring role in their own care: A bibliometric analysis of the on-going literature debate. *Health Expect.* 19 516–526. 10.1111/hex.12299 25369557 PMC5055237

[B37] Mokkink L. B. de Vet H. C. W. Prinsen C. A. C. Patrick D. L. Alonso J. Bouter L. M. (2018). Risk of bias checklist for systematic reviews of patient-reported outcome measures. *Qual. Life Res.* 27 1171–1179. 10.1007/s11136-017-1765-4 29260445 PMC5891552

[B38] Mokkink L. B. Prinsen C. A. Bouter L. M. Vet H. C. Terwee C. B. (2016). The COnsensus-based Standards for the selection of health Measurement INstruments (COSMIN) and how to select an outcome measurement instrument. *Braz. J. Phys. Ther.* 20 105–113. 10.1590/bjpt-rbf.2014.0143 26786084 PMC4900032

[B39] Moreno-Chico C. González-de Paz L. Monforte-Royo C. Arrighi E. Navarro-Rubio M. D. Gallart Fernández-Puebla A. (2017). Adaptation to European Spanish and psychometric properties of the patient activation measure 13 in patients with chronic diseases. *Fam. Pract.* 34 627–634. 10.1093/fampra/cmx022 28379415

[B40] Náfrádi L. Nakamoto K. Schulz P. J. (2017). Is patient empowerment the key to promote adherence? A systematic review of the relationship between self-efficacy, health locus of control and medication adherence. *PLoS One* 12:e0186458. 10.1371/journal.pone.0186458 29040335 PMC5645121

[B41] Ngooi S. S. Lee W. W. Alkureishi M. A. Ukabiala O. Venable L. R. Staisiunas D. D. (2016). Patient perceptions of electronic medical record use by faculty and resident physicians: a mixed methods study. *J. Gen. Intern. Med.* 31 1315–1322. 27400921 10.1007/s11606-016-3774-3 PMC5071284

[B42] Paulo Silva Cunha J. Dias D. (2018). Wearable health devices—vital sign monitoring, systems and technologies. *Sensors* 18:2414. 10.3390/s18082414 PMC6111409 30044415

[B43] Prinsen C. A. C. Mokkink L. B. Bouter L. M. Alonso J. Patrick D. L. de Vet H. C. W. (2018). COSMIN guideline for systematic reviews of patient-reported outcome measures. *Qual. Life Res.* 27 1147–1157. 10.1007/s11136-018-1798-3 29435801 PMC5891568

[B44] Pushparajah D. S. (2018). Making patient engagement a reality. *Patient* 11 1–8. 10.1007/s40271-017-0264-6 28741235 PMC5766722

[B45] Rademakers J. Maindal H. T. Steinsbekk A. Gensichen J. Brenk-Franz K. Hendriks M. (2016). Patient activation in Europe: An international comparison of psychometric properties and patients’ scores on the short form Patient Activation Measure (PAM-13). *BMC Health Serv. Res.* 16:570. 10.1186/s12913-016-1828-1 27729079 PMC5059995

[B46] Schmaderer M. Pozehl B. Hertzog M. Zimmerman L. (2015). Psychometric properties of the patient activation measure in multimorbid hospitalized patients. *J. Nurs. Meas.* 23 128–141. 10.1891/1061-3749.23.3.E128 26673761

[B47] Scholl I. Kriston L. Dirmaier J. Buchholz A. Härter M. (2012). Development and psychometric properties of the Shared Decision Making Questionnaire-physician version (SDM-Q-Doc). *Patient Educ. Couns.* 88, 284–290. 22480628 10.1016/j.pec.2012.03.005

[B48] Serrani Azcurra D. J. (2014). Elders Health Empowerment Scale: Spanish adaptation and psychometric analysis. *Colombia Med. (Cali, Colombia)* 45 179–185. PMC4350384 25767307

[B49] Skolasky R. L. Green A. F. Scharfstein D. Boult C. Reider L. Wegener S. T. (2011). Psychometric properties of the patient activation measure among multimorbid older adults. *Health Serv. Res.* 46 457–478. 10.1111/j.1475-6773.2010.01210.x 21091470 PMC3064914

[B50] Skolasky R. L. Ter Gunne A. F. P. Mohamed A. S. Van Laarhoven C. J. Cohen D. B. (2010). The presentation, incidence, etiology, and treatment of surgical site infections after spinal surgery. *Spine* 35 1323–1328. 20150831 10.1097/BRS.0b013e3181bcde61

[B51] Small N. Bower P. Chew-Graham C. A. Whalley D. Protheroe J. (2013). Patient empowerment in long-term conditions: Development and preliminary testing of a new measure. *BMC Health Serv. Res.* 13:263. 10.1186/1472-6963-13-263 23835131 PMC3725177

[B52] Smoth M. D. Bagian J. P. Bryk A. S. Cassell G. H. Conway J. B. Darling H. B. (2013). “Best care at lower cost: The path to continuously learning health care in America,” in *Committee on the learning health care system in America; Institute of Medicine*, eds Smith M. Saunders R. Stuckhardt L. McGinnis J. M. (Washington, DC: National Academies Press (US). 24901184

[B53] Søgaard M. B. Andresen K. Kristiansen M. (2021). Systematic review of patient-engagement interventions: Potentials for enhancing person-centred care for older patients with multimorbidity. *BMJ Open* 11:e048558. 10.1136/bmjopen-2020-048558 34916309 PMC8679112

[B54] Stepleman L. Rutter M-C. Hibbard J. Johns L. Wright D. Hughes M. (2010). Validation of the patient activation measure in a multiple sclerosis clinic sample and implications for care. *Disabil. Rehabil.* 32 1558–1567. 20590506 10.3109/09638280903567885

[B55] Terwee C. B. Bot S. D. de Boer M. R. van der Windt D. A. Knol D. L. Dekker J. (2007). Quality criteria were proposed for measurement properties of health status questionnaires. *J. Clin. Epidemiol.* 60 34–42. 10.1016/j.jclinepi.2006.03.012 17161752

[B56] Tušek-Bunc K. Petek-Šter M. Šter B. Petek D. Kersnik J. (2014). Validation of the Slovenian version of patient assessment of chronic illness care (PACIC) in patients with coronary heart disease. *Coll. Antropol.* 38 437–444. 25144971

[B57] Usta D. Korkmaz F. Akyar I. Bonanomi A. (2019). Patient health engagement scale (PHE-s): Validity and reliability for Turkish patients with chronic diseases. *Cukurova Universitesi Tip Fakultesi Dergisi* 44 1055–1063. 10.17826/cumj.482420

[B58] Weil A. R. (2016). The patient engagement imperative. *Health Aff. (Millwood).* 35:563. 10.1377/hlthaff.2016.0337 27044951

[B59] Wensing M. van Lieshout J. Jung H. P. Hermsen J. Rosemann T. (2008). The Patients Assessment Chronic Illness Care (PACIC) questionnaire in The Netherlands: A validation study in rural general practice. *BMC Health Serv. Res.* 8:182. 10.1186/1472-6963-8-182 18761749 PMC2538520

[B60] World Health Organization (2016). *Multimorbidity.* Available online at: https://apps.who.int/iris/bitstream/handle/10665/252275/9789241511650-eng.pdf

[B61] Yen H. Y. Lin L. J. (2018). Quality of life in older adults: Benefits from the productive engagement in physical activity. *J. Exerc. Sci. Fit.* 16 49–54. 10.1016/j.jesf.2018.06.001 30662493 PMC6323155

[B62] Zakeri S. Chatterjee P. Konstantas D. Ecer F. (2023). A decision analysis model for material selection using simple ranking process. *Sci. Rep.* 13:8631. 10.1038/s41598-023-35405-z PMC10224978 37244904

[B63] Zeng H. Jiang R. Zhou M. Wu L. Tian B. Zhang Y. (2019). Measuring patient activation in Chinese patients with hypertension and/or diabetes: Reliability and validity of the PAM13. *J. Int. Med. Res.* 47 5967–5976. 10.1177/0300060519868327 31601130 PMC7045661

[B64] Zhang Y. Graffigna G. Bonanomi A. Choi K. C. Barello S. Mao P. (2017). Adaptation and validation of a Chinese version of patient health engagement scale for patients with chronic disease. *Front. Psychol.* 8:104. 10.3389/fpsyg.2017.00104 28220090 PMC5292425

